# Comparative Effects of Riboflavin-UVA and Rose Bengal–Green Light Cross-Linking on Corneal Neovascularization

**DOI:** 10.1167/iovs.67.5.16

**Published:** 2026-05-07

**Authors:** Merve Oral, Duygu Dayanir, Gökce Nur Arik Erol, Mehmet Cüneyt Özmen

**Affiliations:** 1 Department of Ophthalmology, Gazi University School of Medicine, Ankara, Turkey; 2 Department of Histology and Embryology, Gazi University School of Medicine, Ankara, Turkey

**Keywords:** corneal neovascularization (CNV), riboflavin-UVA cross-linking (RF/UVA CXL), rose bengal-green light cross-linking (RB/green light CXL), animal testing

## Abstract

**Purpose:**

The purpose of this study was to compare the effects of riboflavin–ultraviolet A (RF/UVA) cross-linking (CXL) and rose bengal–green light (RB/green light) CXL on corneal neovascularization (CNV) in rat eyes.

**Methods:**

Forty Wistar albino rats underwent CNV induction by corneal suture placement and were randomized into four groups: control, RF/UVA CXL, RB/green light CXL, and green light alone. Treatments were administered on day 3. Anterior segment (AS) photographs and AS-optical coherence tomography (AS-OCT) images were obtained on days 0, 3, 7, and 14, with days 7 and 14 defined as endpoints. Right corneas were analyzed using immunohistochemistry (IHC) for vascular endothelial growth factor (VEGF), lymphatic vessel endothelial hyaluronan receptor (LYVE-1), and CD68, along with hematoxylin & eosin (H&E) staining and TUNEL assay. Left corneas were evaluated with ELISA for VEGF and CD68 levels.

**Results:**

The least CNV area was observed in the RF/UVA group on days 7 and 14 (*P* = 0.002 and *P* < 0.001, respectively). The RB/green light CXL group also demonstrated CNV area than the green light alone group on day 7 (*P* < 0.001). ELISA revealed significantly lower VEGF levels in all treatment groups compared with controls on day 7, whereas this reduction persisted only in the RF/UVA CXL group on day 14 (*P* < 0.001 and *P* = 0.049, respectively). VEGF and LYVE-1 immunostaining were significantly lower in the RF/UVA CXL group compared with the green light group at both time points. CD68 staining was reduced in both CXL groups. TUNEL analysis showed the highest keratocyte apoptotic index in the RF/UVA CXL group, followed by the RB/green light CXL group (both *P* < 0.001).

**Conclusions:**

Both CXL modalities suppressed inflammation and were effective in reducing hemangiogenesis, and lymphangiogenesis; however, the RF/UVA CXL group demonstrated superior overall efficacy than the RB/green light CXL group.

Corneal avascularity is essential for maintaining the transparency and immune privilege of the cornea. This avascular privilege can be disrupted by various corneal disorders and hypoxic diseases, resulting in the ingrowth of pathological blood and lymphatic vessels into the cornea from the limbus, a process known as corneal neovascularization (CNV).^1^ CNV is one of the leading causes of blindness worldwide.^2^

Corneal collagen cross-linking (CXL) with riboflavin and UVA (RF/UVA CXL),^3^ is mainly used to prevent progression of ectatic corneal disorders and to treat bacterial and fungal keratitis.^4^ It has also been explored as a therapeutic option for CNV, targeting vascular endothelial cell invasion of the cornea mediated by collagenases.^5^ Previously, RF/UVA CXL has been reported to increase graft survival when applied to mature corneal vessels, whereas it has been reported to be transiently effective in reducing hemangiogenesis and lymphangiogenesis.^6^^,^^7^

We hypothesize that rose bengal-green light CXL (RB/green light CXL) might be another alternative method to reduce CNV, providing covalent cross-links formed between collagen molecules for increasing corneal stiffness by using green light to activate rose bengal.^8^^,^^9^ Although no study has yet evaluated the effect of RB/green light CXL on CNV, rose bengal-mediated photodynamic therapy (RB-PDT), which is used as a treatment modality for infectious keratitis, has been increasingly investigated.^10^ Its application prior to keratoplasty in infectious keratitis has been associated with significantly improved graft survival.^11^ This beneficial effect may be attributed not only to its antimicrobial properties but also to its potential impact on CNV by the effect of reactive oxygen radicals and apoptosis in the vascular endothelial cells.^12^ In addition, increased stromal stiffness may enhance resistance to collagenase activity and thereby hinder the invasion of blood vessels into the corneal stroma.^8^ In addition, green light has been previously used to regress corneal vessels.^13^ Green light used in rose bengal CXL might also help alleviate CNV in addition to its CXL effect in tissue. The aim of this study was to evaluate the comparative effects of RF/UVA CXL and RB/green light CXL on CNV in rateyes.

## Methods

**Figure 1.**
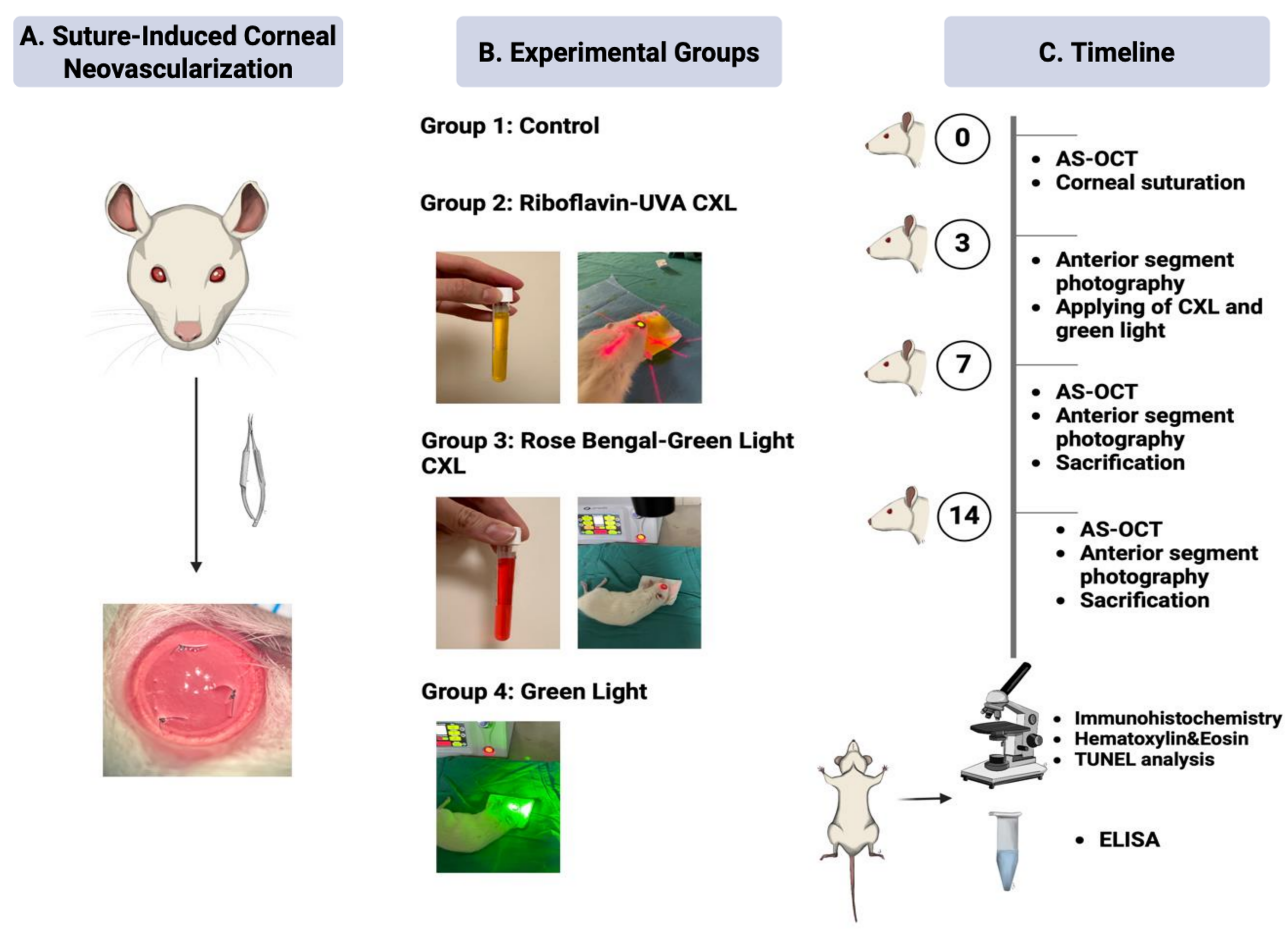
Graphical abstract of the study design. (**A**) Suture induced model. (**B**) Experimental groups. (**C**) Timeline of the study.

All procedures complied with the ARVO Statement for the Use of Animals in Ophthalmic and Vision Research. The study was conducted in accordance with a protocol approved by Gazi University Animal Experiments Local Ethics Committee (G.U.ET-23.014) and followed the guidelines of the laboratory animal care. Forty female, albino Wistar rats, 8 to 10 weeks old (weight = 200 to 300*g*) were used. Animals were anesthetized with an intraperitoneal injection of ketamine hydrochloride (45 mg/kg) and xylazine hydrochloride (5 mg/kg), supplemented with topical proparacaine hydrochloride 0.5% (Alcaine; Alcon Laboratories, Inc., Fort Worth, TX, USA). CNV was induced by suture placement as previously described.^14^ In short, 3 single 10-0 nylon sutures were placed through the corneal stroma 1.5 to 2 mm anterior to the limbus at 4, 8, and 12 o’clock positions in both eyes of each rat (Fig. 1A). The knots and suture ends were left exposed. Moxifloxacin 0.5% (Moxai, Abdi Ibrahim, Turkiye) drops were instilled twice daily for 1 week. The animals were randomly divided into four groups using a computer-generated simple randomization method (1:1:1:1 allocation ratio, Excel generated random sequence): (1) the control group, (2) the RF/UVA CXL group, (3) the RB/green light CXL group, and (4) the green light only group (Fig. 1B). Treatments were administered on day 3 after confirming the clinical onset of CNV in all eyes. The control, RF/UV-A, and RB/green light CXL groups each included 10 rats. In the green light only group, one rat died due to a general anesthesia complication; therefore, 9 rats were ultimately included in this group. On day 7, there were 5 rats from each group that were euthanized, and the remaining rats were euthanized on day 14. Subsequently, the corneas were dissected from the corneoscleral junction. For histopathological evaluation, corneal samples obtained from the right eyes of each euthanized animal were fixed in 10% neutral buffered formalin. For ELISA quantification, corneas from the left eyes were placed in tubes and snap-frozen in liquid nitrogen. Central corneal thickness (CCT) was measured using anterior segment optical coherence tomography (AS-OCT; MS-39; CSO, Florence, Italy) on days 0, 7, and 14 (Fig. 1C).

### Treatment Protocols

In group 2, the RF/UVA CXL procedures were conducted according to previous research, which determined the safety and efficacy of CXL on rat eyes.^15^ After scraping the corneal epithelium in the eyes gently, 0.22% riboflavin solution (10 mg riboflavin in 4.5 mL 20% dextran T-500 solution) was instilled every 3 minutes for 30 minutes, and UVA was delivered at a dose of 9 mW/cm^2^ for 4 minutes (total energy dose = 2.16 J/cm^2^).

For group 3, as there were no reports in the literature on the application of RB/green light CXL to the eyes of rats, we decided to carry out a preliminary experiment using both eyes of one rat to determine the safety for the corneal endothelium. Following mechanical debridement of the epithelium, 0.1% rose bengal solution (0.1 gram rose bengal in 100 mL distilled water) was applied to the rat cornea every 15 seconds for 2 minutes, followed by 532 nm green light (Fenix 532-S, Omesis, Turkiye) with a power of 250 mW for 200 seconds, 0.1% rose bengal drops instilled every 15 seconds for 30 seconds, followed by continuous mode irradiation for 200 seconds (total energy dose = 100 J/cm^2^).^16^ Histopathological evaluation on day 4 showed corneal endothelial integrity. Once safety was confirmed, RB/green light CXL procedures were performed at predetermined doses on group 3. A green light (200 seconds at 250 mW 532 nm) was applied to group 4 without the use of a photosensitizer to analyze the actual CXL effect of rose bengal.^13^ Except for the installation of rose bengal, all other procedures were performed in the same manner as in group 3. Balanced salt solution (BSS; Ocrosol, Polifarma, Turkiye) was instilled on the corneal surface every 60 seconds to prevent corneal drying during irradiation.

### Analysis of CNV Area

Anterior segment photographs were obtained at days 0 (suture implantation), 3, 7, and 14 using a digital camera mounted on a light microscope (Leica S8APO, Wetzlar, Germany) at 1.25 × magnification. Three central images were taken from each cornea. To avoid confusion between iris vessels and CNV in albino rats, one drop of 0.5% tropicamide (Tropamid, Bilim Pharmaceuticals, Turkey) was instilled 15 minutes before imaging.^17^

The CNV area was calculated using ImageJ software (Wayne Rasband at Research Services Branch, National Institutes of Health, Bethesda, MD, USA) with methods described before.^18^ For analysis, the color image was split into red, green, and blue channels, and the green channel was selected to enhance vessel contrast. After manually optimizing contrast and shading, the image was converted to 8-bit color and was adjusted to include the dark vessels and exclude the brighter background. The area defined by the threshold setting was calculated and normalized to the total region of interest (expressed as a percentage covered by blood vessels). Measurements were made by two masked researchers (authors M.O. and B.A.), and the mean of these measurements was used for statistical analysis.

### Histological Analysis

Corneas fixed in phosphate-buffered 10% formaldehyde were dehydrated in graded ethanol and embedded in paraffin. Five-micrometer sections were stained with hematoxylin and eosin (H&E) for examination under light microscopy (Leica). The findings were evaluated by 2 masked histologists (authors D.D. and G.A.) who evaluated 5 fields per sample at × 40 magnification. Inflammation was graded by polymorphonuclear leukocyte count (0 = none; 1 = 1–5 cells; 2 = 6–15 cells; 3 = 16–25 cells; and 4 = more than 25 cells).^19^ The number of keratocytes was counted in the upper 25% of the stroma.^20^

### Immunohistochemical Analysis

All sections were deparaffinized, passed through decreasing alcohols, and incubated in citrate buffer (pH = 6.0) and 3% hydrogen peroxide. After blocking (Ultra V block, Genemed Biotechnology, South San Francisco, CA, USA), tissues were incubated overnight at 4°C with primary antibodies: anti-CD68 (dilution 1:200; Bioss Inc., Woburn, MA, USA), anti-LYVE-1 (dilution 1:200; Bioss), and anti-VEGF (dilution 1:25; Bioss). Sections were then incubated with secondary antibody biotinylated goat anti-polyvalent (Thermo-Scientific, Waltham, MA, USA), followed by streptavidin peroxidase and diaminobenzidine (DAB; Lab Vision, Fremont, CA, USA) complex. Mayer's hematoxylin served as the counterstaining. Photomicrographs were taken with a light microscope (Leica DCM 4000). Immunostaining for VEGF, LYVE-1, and CD68 was assessed by masked observers (authors D.D. and G.A.) using a scoring system for staining intensity: No (0), very weak (1), moderate (2), strong (3), and very strong (4) expressions in the immunohistochemical examinations. The percentage of positive cells was graded as follows. Grade 0 indicated <5% positive cells, grade 1 indicated 6% to 15%, grade 2 indicated 16% to 50%, grade 3 indicated 51% to 80%, and grade 4 indicated >80% positive cells.^21^

### TUNEL Analysis

Keratocyte apoptosis was evaluated by terminal deoxynucleotidyl transferase dUTP nick end labeling (TUNEL) with an apoptosis kit (Elabscience, Houston, TX, USA) on frozen sections following the manufacturer's instructions. TUNEL-positive cells were visualized with DAB and counterstained with Mayer's hematoxylin. Photomicrographs were taken using a light microscope (Leica DM 1000). Apoptosis was quantified by analyzing 5 randomly selected stromal fields at 400 × magnification, and the TUNEL index (%) was calculated as the ratio of TUNEL-positive cells divided by the total number of cells.^22^

### ELISA Analysis

Corneal tissues were stored at −80°C and mechanically homogenized in phosphate-buffered saline (pH 7.4). Homogenates were centrifuged at 5000 rpm for 10 minutes, and the supernatants were collected. VEGF and CD68 concentrations were measured by sandwich enzyme-linked immunosorbent assay (ELISA) kits (BT Lab; VEGF: E0659Ra, CD68: E1384Ra, Shanghai, China) following the manufacturer’s instructions. Absorbance was read at 450 nm. VEGF levels were calculated as ng/L, CD68 levels as ng/mL per mg of tissue.

### Statistical Analysis

Statistical analyses were performed using IBM SPSS Statistics 27.0 (IBM Corp., Armonk, NY, USA). Descriptive statistics and frequency tables were generated. Normality was assessed using the Kolmogorov–Smirnov test. For normally distributed variables, ANOVA, paired *t*-tests, and repeated-measures analyses were used; for non-normally distributed data, Kruskal–Wallis, Wilcoxon, and Friedman tests were applied. For two-sided hypothesis testing, statistical significance was set at *P* < 0.05.

## Results

### Clinical Findings

The CCT of all animals was 142.3 ± 15 µm at baseline. At the consecutive visit on day 7, CCT was higher in all treatment groups compared to the control group (*P* < 0.001). On day 14, CCT was similar between the groups (*P* > 0.05; Supplementary Table S1).

**Figure 2.**
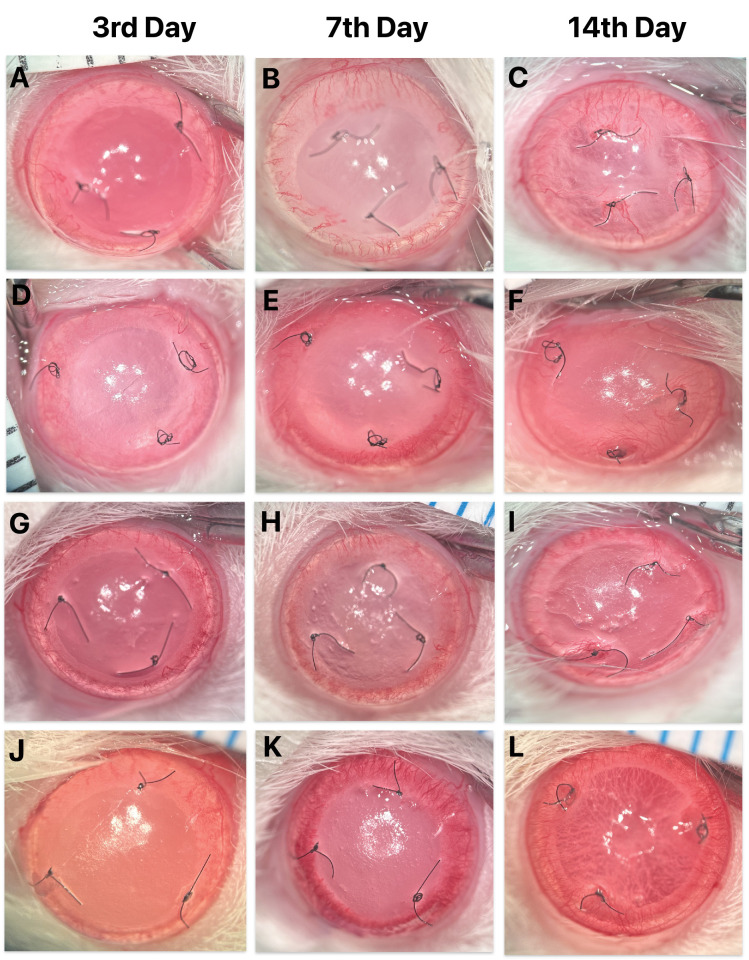
Representative anterior segment photographs of eyes from each treatment group at days 3, 7, and 14. (**A**, **B**, **C**) control group; (**D**, **E**, **F**) riboflavin-UVA CXL group; (**G**, **H**, **I**) rose bengal–green light group; (**J**, **K**, **L**) green light only group. No apparent differences in corneal neovascularization (CNV) were observed among groups at day 3. At days 7 and 14, the CNV area was lower in the riboflavin-UVA CXL group (**E** and **F**) compared with the other groups.

**Figure 3.**
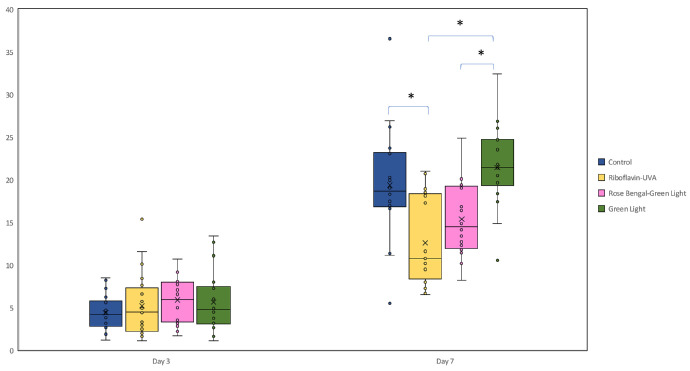
Comparison of corneal neovascularization (CNV) area among treatment groups at days 3 and 7. At day 7, the RB/green light group showed a lower CNV area than the green light only group, whereas the RF-UVA CXL group demonstrated the lowest CNV area among all groups.

A comparison of the CNV area, including a total of 38 eyes examined on days 3, 7, and 14 revealed significant differences (Fig. 2). There was no significant difference on day 3 among the groups (*P* > 0.05). The CNV area at day 7 and day 14 was significantly lower in the RF/UVA CXL group compared with other groups (*P* = 0.002 and *P* < 0.001, respectively). The vascularized area increased on days 7 and 14 compared to day 3 in all groups (*P* < 0.001); the difference between days 7 and 14 within the groups was not significant. When comparing the 76 eyes on days 3 and 7, the RF/UV-A CXL group was less vascularized compared with both the control and green light only groups (*P* < 0.001). Additionally, the RB/green light CXL group had significantly less CNV area than the green light only group (*P* < 0.001; Fig. 3).

### Histological Findings

**Figure 4.**
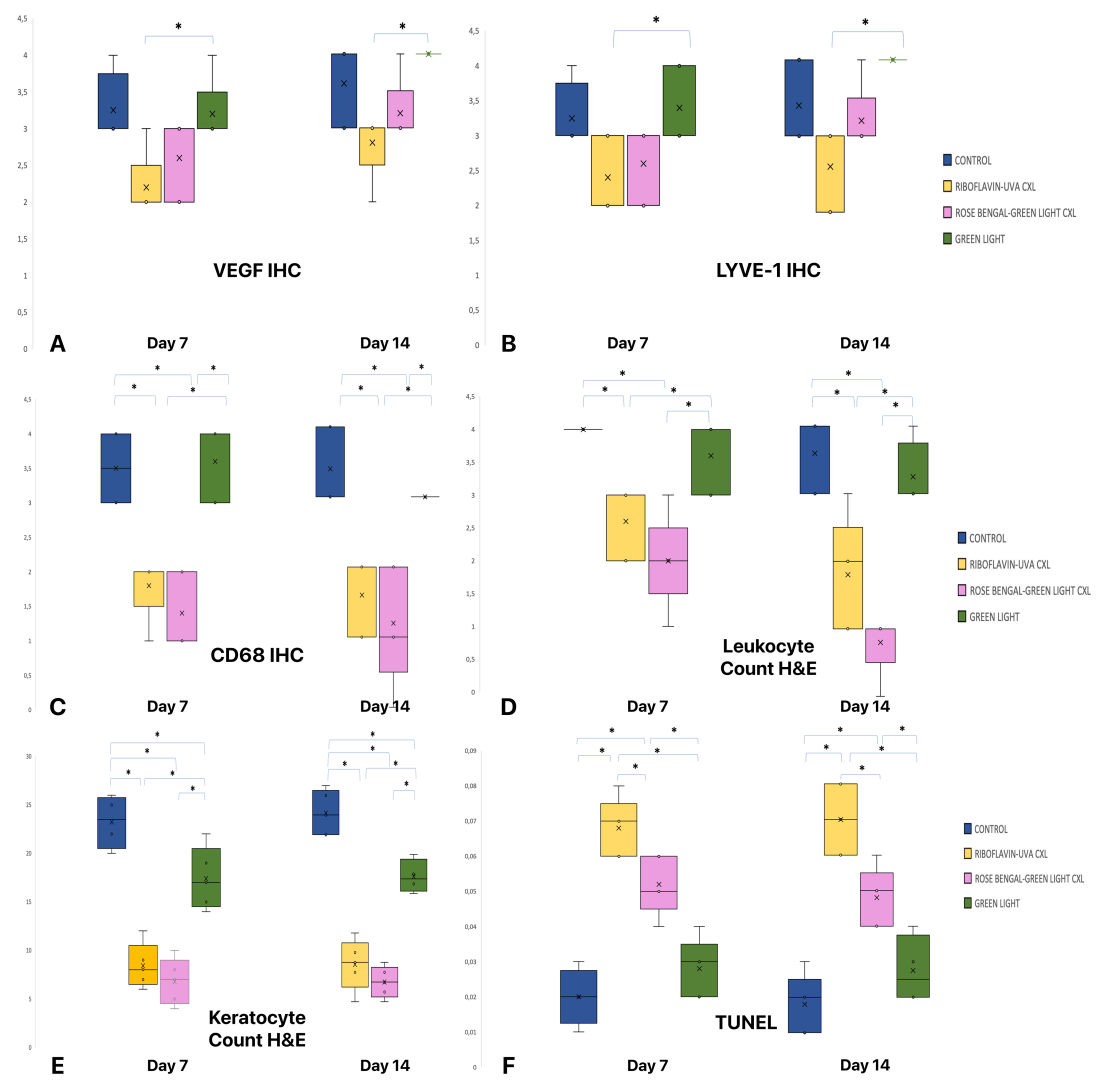
VEGF immunohistochemistry (IHC) scores in groups on days 7 and 14 (**A**), LYVE-1 IHC scores on days 7 and 14 (**B**), CD68 IHC scores on days 7 and 14 (**C**). Leukocyte counts (**D**) and keratocyte counts (**E**) between the groups on the 7th and 14th days by hematoxylin and eosin staining (H&E). TUNEL analysis between groups on days 7 and 14 (**F**).

**Figure 5.**
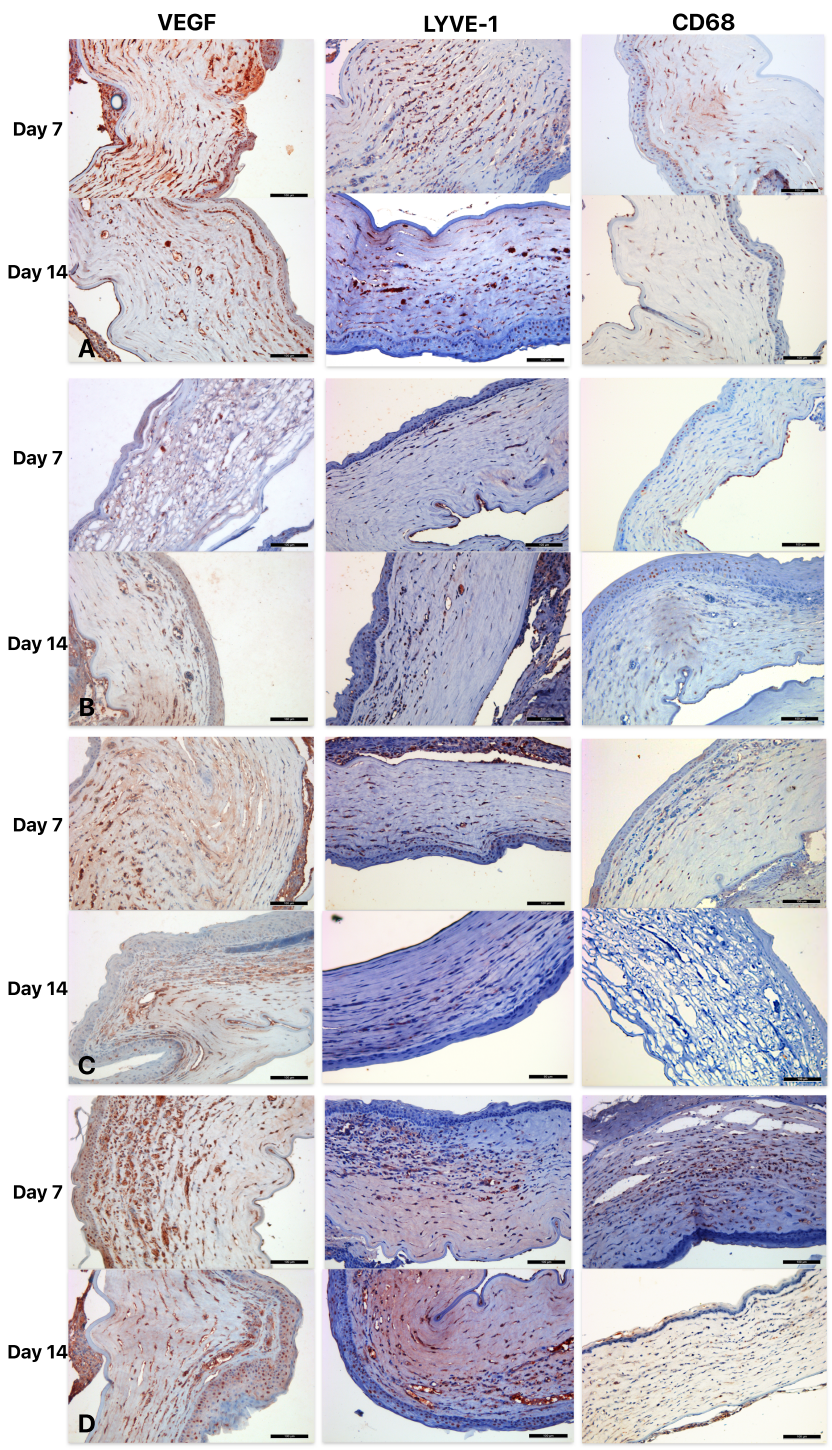
Photomicrographs of VEGF, LYVE-1, and CD-68 immunohistochemical staining in control (**A**), riboflavin-UVA CXL (**B**), RB/green light CXL (**C**) and the green light only group (**D**) on days 7 and 14. Brown staining indicates positive cells. (*Scale bar* = 100 µm).

On immunohistochemical examination, VEGF staining demonstrated significantly lower VEGF scores in the RF/UVA CXL group compared with the green light only group on both days 7 and 14 (*P* = 0.032 and *P* = 0.016, respectively; Fig. 4A). Similarly, LYVE-1 expression was lower in RF/UVA CXL group compared to green light only group at both time points (*P* = 0.046 and *P* = 0.016, respectively; Fig. 4B). The CD68 staining scores evaluated on days 7 and 14 were significantly lower in both CXL groups compared to the control and green light only groups (*P* = 0.002 and *P* = 0.002, respectively; Figs. 4C, 5).

Keratocyte and leukocytic cells were counted by H&E staining. Leukocytic infiltration was significantly higher in the control and green light only groups compared with the cross-linked groups both on days 7 and 14 (*P* = 0.003 and *P* = 0.002, respectively; Fig. 4D). Similarly, whereas the most keratocyte counts were evaluated in the control group, both cross-linked groups included fewer keratocytes than the green light only group (*P* < 0.001 and p < 0.001, respectively). No significant difference was observed between the cross-linked groups (Fig. 4E).

The highest apoptotic index of keratocytes was examined in the RF/UVA CXL groups on days 7 and 14 (*P* < 0.001 and *P* < 0.001, respectively). The RB/green light CXL group exhibited significantly higher apoptosis compared to the green light only and control groups on days 7 and 14 (*P* < 0.001 and *P* < 0.001, respectively; Fig. 4F).

### ELISA Analysis

VEGF levels of both CXL groups and the green light only group on day 7 were significantly lower than the control group (*P* < 0.001). On day 14, only VEGF levels between RF/UVA CXL and the control group remained statistically significant (*P* = 0.049). CD68 levels among the groups on day 7 were not different (*P* > 0.05). However, CD68 levels in the RB/green light CXL group were significantly lower compared to the control group on day 14 (*P* = 0.013) (see the Table).

**Table.** ELISA VEGF and CD68 Levels in Groups on Days 7 and 14

	Control^1^ (*n* = 10)	Riboflavin-UVA^2^ (*n* = 10)	Rose Bengal-Green Light^3^ (*n* = 10)	Green Light^4^ (*n* = 9)		
	Mean ± SD	Mean ± SD	Mean ± SD	Mean ± SD	*P* Value^*^	Post Hoc Analysis [Groups]^‡^
ELISA VEGF, ng/L
Day 7	1095.92 ± 278.80	446.46 ± 51.49	408.46 ± 93.79	404.15 ± 87.21	***P*** **< 0.001**	***P*** **< 0.001 [1, 2]**
						***P*** **< 0.001 [1–3]**
						***P*** **< 0.001 [1–4]**
						*P* = 0.974 [2, 3]
						*P =* 0.965 [2–4]
						*P =* 1.00 [3, 4]
Day 14	1156.46 ± 436.27	699.53 ± 115.60	885.69 ± 63.09	1091.31 ± 187.49	***P* = 0.047**	***P*** **= 0.049 [1, 2]**
						*P* = 0.351 [1–3]
						*P =* 0.979 [1–4]
						*P* = 0.649 [2, 3]
						*P* = 0.133 [2–4]
						*P* = 0.620 [3, 4]
*P* value^†^	*P* = 0.818	***P* = 0.002**	***P* < 0.001**	***P* < 0.001**		
ELISA CD68, ng/mL
Day 7	18.31 ± 4.31	18.54 ± 4.17	17.32 ± 1.89	15.21 ± 3.41	*P* = 0.462	
Day 14	9.56 ± 1.24	6.39 ± 3.21	3.43 ± 3.01	4.68 ± 2.97	***P*** **= 0.017**	***P*** **= 0.013 [1**–**3]**
						*P* = 0.290 [1, 2]
						*P* = 0.073 [1–4]
						*P* = 0.346 [2, 3]
						*P* = 0.787 [2–4]
						*P* = 0.899 [3, 4]
*P* value^†^	***P* = 0.003**	***P* < 0.001**	***P* < 0.001**	***P* = 0.002**		

SD, standard deviation.

The *P* values in bold represent statistical significance.

* Differences among the four groups at each examination (1-way ANOVA).

† Differences among all follow-up examinations within each group (Paired-samples *t*-test).

‡ Between group differences at each examination (Tukey post hoc test).

## Discussion

This study is the first to assess the impact of RB/green light CXL on CNV. The present study showed RB/green light CXL is effective in ameliorating CNV.

The timing of treatment administration was determined based on previous studies, which showed that hemangiogenesis begins within hours and lymphangiogenesis within approximately 3 days after injury; hence, in this study, we performed CXL treatments on day 3 following clinical confirmation of CNV.^23^^,^^24^ As neovascularization typically peaks around day 14 and subsequently regresses,^23^ the evaluation endpoints were set at 7 and 14 days to accurately assess treatment efficacy.

To assess the safety of endothelial cell function, CCT was analyzed. Previously, CCT was shown to have no difference between RF/UVA and RB/green light CXL, with a transient increase on day 2 returning to baseline by day 4.^12^ Similarly, in our study, CCT in the CXL groups showed a significant increase compared to the control group on day 7, but decreased thereafter, and the difference between groups disappeared on day 14.

The impact of RF/UVA CXL on CNV was first investigated on suture-induced CNV in murine eyes by Bock et al.^5^ In their study, CXL was applied at a dose of 5.4 J/cm^2^ on day 14 after suturing, and a negative correlation between corneal stromal depth and vascular area was reported. The optimal dose for CXL on rat eyes was determined in the study of Zhu et al., and found to be safe up to 2.7 J/cm^2^. Meanwhile, a transient decline in the endothelium at 2.7 J/cm^2^ was shown, making 2.16 J/cm^2^ more reliable in these eyes.^15^

Previous studies have evaluated the effect of RF/UVA CXL on blood and lymphatic vessels. It was shown that both types of vessels regressed compared to the control after CXL treatment.^6^ In our study, the CNV area was significantly lower in the RF/UVA CXL and RB/green light CXL groups on days 7 and 14. This result supports the effect of RB/green light CXL on vascularization, which had not been evaluated previously.

VEGF, the main cytokine involved in angiogenesis in the tissue, was analyzed by ELISA and immunohistochemistry. ELISA VEGF levels were significantly lower in both CXL and green light groups compared to the control group on day 7, whereas the difference remained significantly lower in the RF/UVA CXL group on day 14. Although the RB/Green light group showed lower VEGF levels compared to the control group on day 7, this difference did not persist until day 14. Immunohistochemical staining of VEGF was also lower in the RF/UVA CXL group at both time points, in parallel with LYVE-1, which is an indicator of lymphangiogenesis. These results, which were similar to ours, were also shown in the study by Zhu et al.^7^ When VEGFR2, VEGFR3, VEGFC, and LYVE1 were observed as transient decreases in the RF/UVA CXL group, this was consistent with their findings by PCR analysis.

Inflammation was evaluated through CD68 levels by ELISA and immunohistochemical staining, and also polymorphonuclear leukocyte count by H&E. In the aforementioned study by Zhu et al., CD45 leukocyte and CD68 macrophage staining was also lower in the RF/UVA CXL group on day 7, consistent with vascularization.^7^ Along with studies of evaluating vascularization after alkali burn, it was observed that CD68 macrophages were less in the RF/UVA CXL groups compared to the control group.^25^^,^^26^ Therefore, reduced inflammation in the CXL groups might explain the lower degree of vascularization in these groups. Our results of immunohistochemistry and H&E staining were significantly lower in both CXL groups on days 7 and 14. ELISA showed the lowest CD68 levels in the RB/green light CXL group on day 14 compared with all groups.

In a study comparing the wound healing responses of two CXL methods, keratocyte depletion was reported in the anterior and mid-stroma after RF/UVA CXL, but only in the anterior stroma in the RB/green light CXL.^12^ Because rose bengal binds collagen more tightly, its CXL effect is known to be more superficial and therefore considered safer in thin corneas.^9^ In our study, a similar effect was observed, and keratocyte apoptosis was highest in the RF/UVA CXL group on TUNEL analysis, followed by RB/green light CXL. This finding was further supported by reduced keratocyte counts on H&E staining. Although we evaluated only keratocyte apoptosis using TUNEL analysis, this finding may be associated with vascular regression, which has been reported as an underlying mechanism in the study by Hou et al., demonstrating regression of corneal blood and lymphatic vessels through apoptosis of vascular endothelial cells.^6^

One of the main limitations of our study is the structural differences between the rat and human corneas, which may influence the response to CXL.^27^ In addition, the regenerative capacity of rat endothelial cells limits comparability with the human cornea when evaluating inflammatory responses. Nevertheless, the effects of RF/UVA CXL have also been demonstrated in clinical studies, showing that treatment of the recipient cornea with CXL may induce regression of corneal vessels at the time of keratoplasty.^28^^,^^29^ Although the long-term durability of this angioregressive effect remains to be fully elucidated,^30^ current evidence suggests that RF/UVA CXL yields favorable outcomes. Another limitation is the differences in the energy doses applied in RB/green light studies. In previously reported RB/green light CXL studies, the applied energy doses generally ranged from 50 to 150 J/cm^2^,^8^ whereas much lower doses (e.g. 5.4 J/cm^2^) have been preferred in studies investigating infectious keratitis.^31^^,^^32^ Although we have confirmed the safety of the applied dose in a preliminary experiment, the fact that RB/green light CXL has been shown to be effective at substantially lower energy levels in the literature indicates that further studies are required to determine the optimal dose and irradiation duration for inhibiting CNV.^32^^,^^33^ Finally, as both CXL modalities demonstrate progressively increasing effects over time, longer follow-up periods are required to fully evaluate their impact on CNV.

In conclusion, whereas RF/UVA CXL was shown to effectively inhibit CNV, this study demonstrates for the first time that RB/green light CXL is also effective in suppressing angiogenesis. The reduced inflammatory response observed in cross-linked corneas may explain the lower degree of vascularization, as inflammation is a key driver of new vessel formation.

## Supplementary Material

Supplement 1

## References

[1] Beebe DC . Maintaining transparency: a review of the developmental physiology and pathophysiology of two avascular tissues. *Semin Cell Dev Biol*. 2008; 19(2): 125–133. 17920963 10.1016/j.semcdb.2007.08.014 PMC2276117

[2] Chang JH, Garg NK, Lunde E, Han KY, Jain S, Azar DT. Corneal neovascularization: an anti-VEGF therapy review. *Surv Ophthalmol*. 2012; 57(5): 415–429. 22898649 10.1016/j.survophthal.2012.01.007 PMC3709023

[3] Spoerl E, Huhle M, Seiler T. Induction of cross-links in corneal tissue. *Exp Eye Res*. 1998; 66(1): 97–103. 9533835 10.1006/exer.1997.0410

[4] Ting DSJ, Henein C, Said DG, Dua HS. Photoactivated chromophore for infectious keratitis - corneal cross-linking (PACK-CXL): a systematic review and meta-analysis. *Ocul Surf*. 2019; 17(4): 624–634. 31401338 10.1016/j.jtos.2019.08.006

[5] Bock F, Tóth G, Szentmary N, Bucher F, Cursiefen C. Corneal crosslinking with ultraviolet-A and riboflavin in a murine model of corneal neovascularisation. *Invest Ophthalmol Vis Sci*. 2015; 56(7): 4517–4517.

[6] Hou Y, Le VNH, Tóth G, et al. UV light crosslinking regresses mature corneal blood and lymphatic vessels and promotes subsequent high-risk corneal transplant survival. *Am J Transplant*. 2018; 18(12): 2873–2884. 29673063 10.1111/ajt.14874 PMC6282984

[7] Zhu Y, Li L, Reinach PS, et al. Corneal collagen cross-linking with riboflavin and UVA regulates hemangiogenesis and lymphangiogenesis in rats. *Invest Ophthalmol Vis Sci*. 2018; 59(8): 3702–3712. 30029257 10.1167/iovs.17-23036

[8] Cherfan D, Verter EE, Melki S, et al. Collagen cross-linking using rose bengal and green light to increase corneal stiffness. *Invest Ophthalmol Vis Sci*. 2013; 54(5): 3426–3433. 23599326 10.1167/iovs.12-11509 PMC4597485

[9] Bekesi N, Kochevar IE, Marcos S. Corneal biomechanical response following collagen cross-linking with rose bengal-green light and riboflavin-UVA. *Invest Ophthalmol Vis Sci*. 2016; 57(3): 992–1001. 26968733 10.1167/iovs.15-18689

[10] Atalay HT, Uysal BS, Sarzhanov F, et al. Rose bengal-mediated photodynamic antimicrobial treatment of acanthamoeba keratitis. *Curr Eye Res*. 2020; 45(10): 1205–1210. 32065854 10.1080/02713683.2020.1731830

[11] Sepulveda-Beltran PA, Levine H, Altamirano DS, et al. Rose bengal photodynamic antimicrobial therapy: a review of the intermediate-term clinical and surgical outcomes. *Am J Ophthalmol*. 2022; 243: 125–134. 35952754 10.1016/j.ajo.2022.08.004

[12] Lorenzo-Martín E, Gallego-Muñoz P, Ibares-Frías L, et al. Rose bengal and green light versus riboflavin-UVA cross-linking: corneal wound repair response. *Invest Ophthalmol Vis Sci*. 2018; 59(12): 4821–4830. 30347076 10.1167/iovs.18-24881

[13] Reed JW, Fromer C, Klintworth GK. Induced corneal vascularization remission with argon laser therapy. *Arch Ophthalmol*. 1975; 93(10): 1017–1019. 1237281 10.1001/archopht.1975.01010020797012

[14] Peebo BB, Fagerholm P, Traneus-Röckert C, Lagali N. Cellular-level characterization of lymph vessels in live, unlabeled corneas by in vivo confocal microscopy. *Invest Ophthalmol Vis Sci*. 2010; 51(2): 830–835. 19797212 10.1167/iovs.09-4407

[15] Zhu Y, Reinach PS, Zhu H, et al. High-intensity corneal collagen crosslinking with riboflavin and UVA in rat cornea. *PLoS One*. 2017; 12(6): e0179580. 28644862 10.1371/journal.pone.0179580 PMC5482453

[16] Wang T, Zhu L, Zhu J, et al. Subacute effects of rose bengal/green light cross linking on rabbit thin corneal stability and safety. *Lasers Surg Med*. 2018; 50(4): 324–332. 29095506 10.1002/lsm.22762

[17] Geng Y, Greenberg KP, Wolfe R, et al. In vivo imaging of microscopic structures in the rat retina. *Invest Ophthalmol Vis Sci*. 2009; 50(12): 5872–5879. 19578019 10.1167/iovs.09-3675 PMC2873188

[18] Cursiefen C, Bock F, Horn FK, et al. GS-101 antisense oligonucleotide eye drops inhibit corneal neovascularization: interim results of a randomized phase II trial. *Ophthalmology*. 2009; 116(9): 1630–1637. 19643487 10.1016/j.ophtha.2009.04.016

[19] Arikan S, Karaca T, Ertekin YH, et al. Effect of topically applied azithromycin on corneal epithelial and endothelial apoptosis in a rat model of corneal alkali burn. *Cornea*. 2016; 35(4): 543–549. 26751994 10.1097/ICO.0000000000000730

[20] Zhu H, Alt C, Webb RH, Melki S, Kochevar IE. Corneal crosslinking with rose bengal and green light: efficacy and safety evaluation. *Cornea*. 2016; 35(9): 1234–1241. 27362877 10.1097/ICO.0000000000000916

[21] Gocmez SS, Yazir Y, Sahin D, Karadenizli S, Utkan T. The effect of a selective neuronal nitric oxide synthase inhibitor 3-bromo 7-nitroindazole on spatial learning and memory in rats. *Pharmacol Biochem Behav*. 2015; 131: 19–25. 25636602 10.1016/j.pbb.2015.01.013

[22] Zhou M, Liu L, Wang W, et al. Role of licochalcone C in cardioprotection against ischemia/reperfusion injury of isolated rat heart via antioxidant, anti-inflammatory, and anti-apoptotic activities. *Life Sci*. 2015; 132: 27–33. 25921769 10.1016/j.lfs.2015.04.008

[23] Cursiefen C, Maruyama K, Jackson DG, Streilein JW, Kruse FE. Time course of angiogenesis and lymphangiogenesis after brief corneal inflammation. *Cornea*. 2006; 25(4): 443–447. 16670483 10.1097/01.ico.0000183485.85636.ff

[24] Ling S, Lin H, Liang L, et al. Development of new lymphatic vessels in alkali-burned corneas. *Acta Ophthalmol*. 2009; 87(3): 315–322. 18811642 10.1111/j.1755-3768.2008.01349.x

[25] Subasi S, Altintas O, Yardimoglu M, et al. Comparison of collagen cross-linking and amniotic membrane transplantation in an experimental alkali burn rabbit model. *Cornea*. 2017; 36(9): 1106–1115. 28704317 10.1097/ICO.0000000000001276

[26] Kesim E, Pirhan D, Yardimoglu Yilmaz M, et al. Comparative analysis of matrix-regenerating agent and corneal cross-linking in an experimental alkali burn rabbit model. *Curr Eye Res*. 2022; 47(2): 187–195. 34435926 10.1080/02713683.2021.1971722

[27] Meek KM, Boote C. The use of X-ray scattering techniques to quantify the orientation and distribution of collagen in the corneal stroma. *Prog Retin Eye Res*. 2009; 28(5): 369–392. 19577657 10.1016/j.preteyeres.2009.06.005

[28] Schaub F, Hou Y, Zhang W, Bock F, Hos D, Cursiefen C. Corneal crosslinking to regress pathologic corneal neovascularization before high-risk keratoplasty. *Cornea*. 2021; 40(2): 147–155. 33395116 10.1097/ICO.0000000000002406

[29] Price FW Jr, Tefasse Z, Frances KD, Feng MT, Gang A, Price MO. Assessment of corneal crosslinking for the treatment of corneal neovascularization with and without associated infection. *Cornea*. 2026; 45(4): 450–456. 40172865 10.1097/ICO.0000000000003869

[30] Wiedemann J, Hos D, Limburg E, et al. UV light-mediated corneal crosslinking as (lymph)angioregressive pretreatment to promote graft survival after subsequent high-risk corneal transplantation (CrossCornealVision): protocol for a multicenter, randomized controlled trial. *Trials*. 2024; 25(1): 169. 38448965 10.1186/s13063-024-08011-1 PMC10916195

[31] Martinez JD, Arrieta E, Naranjo A, et al. Rose bengal photodynamic antimicrobial therapy: a pilot safety study. *Cornea*. 2021; 40(8): 1036–1043. 34190718 10.1097/ICO.0000000000002717 PMC8504203

[32] Hafezi NL, Aydemir ME, Lu NJ, Torres-Netto EA, Hillen M, Koppen C. Effect of accelerated high-fluence riboflavin and rose bengal-mediated corneal cross-linking on resistance to enzymatic digestion. *BMC Ophthalmol*. 2024; 24(1): 37. 38267904 10.1186/s12886-024-03293-0 PMC10809678

[33] Yesilirmak N, Saritas O. Comparison of corneal biomechanical efficacy between rose bengal-green light and riboflavin-UVA crosslinking. *Curr Eye Res*. 2024; 49(9): 942–948. 38747449 10.1080/02713683.2024.2353267

