# Supplementary material for: Comparative Effects of Riboflavin-UVA and Rose Bengal–Green Light Cross-Linking on Corneal Neovascularization
Source: Invest Ophthalmol Vis Sci. 2026 May 7;67(5):16. doi: 10.1167/iovs.67.5.16 (PMC13170724; doi:10.1167/iovs.67.5.16)
Supplement: Supplement 1 [file iovs-67-5-16_s001.pdf]

Supplementary Table-S1: Central corneal thicknesses (µm) on days 0, 7 and 14 among the groups.

	Control[1] (n=20)	Riboflavin-UVA[2] (n=20)	Rose Bengal-Green Light (n=20)	Green Light[4] (n=18)	p-value*	Post-hoc analysis [groups]‡
	Mean ± SD	Mean ± SD	Mean ± SD	Mean ± SD		
Day 0	146.20± 16.06	144.90± 11.47	146.10± 15.55	134.87± 9.93	p = 0.277	
Day 7	229.40± 32.68	350.80± 75.64	357.90± 56.73	357.50± 95.48	**p <0.001**	**p<0.001[1-2]**<br>**p<0.001[1-3]**<br>**p<0.001[1-4]**<br>p= 0.456 [2-3]<br>p= 0.090 [2-4]<br>p= 0.781 [3-4]
Day 14	255.40± 42.44	257.70± 60.13	287.80± 81.66	272.00± 87.06	p = 0.706	
p-value†	**p < 0.001**	**p < 0.001**	**p < 0.001**	**p < 0.001**		
Post-hoc analysis [days]§	**p < 0.001 [0-7]**<br>**p < 0.001 [0-14]**<br>p = 0.160 [7-14]	**p < 0.001 [0-7]**<br>**p = 0.001 [0-14]**	**p < 0.001 [0-7]**<br>**p = 0.002 [0-14]**<br>**p = 0.02 [7-14]**	**p = 0.01 [0-7]**<br>**p = 0.012 [0-14]**<br>**p = 0.031 [7-14]**		

SD; standard deviation
**Differences among the four groups at each examination (One-way ANOVA test).*
†Differences among all follow-up examinations within each group (Repeated Measures test)
*§Within group differences between paired examination (**Bonferroni-adjusted paired comparisons)**.*
*‡Between group differences at each examination (**Tukey post hoc test)**.*
